# Performance optimization of Co/Zn-ZIF-8/PDMS mixed-matrix membranes based on tubular ceramic carriers for ethanol recovery *via* pervaporation

**DOI:** 10.1039/d5ra01301a

**Published:** 2025-06-11

**Authors:** Bo Yuan, Jiaxuan Gou, Yuzhuang Li, Ke Li, Qun Liu, Yu Zhang

**Affiliations:** a College of Petrochemical Engineering, Jilin Institute of Chemical Technology Jilin 132000 PR China yb19880613@163.com +86 432 62185157

## Abstract

In the present investigation, highly hydrophobic Zn-ZIF-8 nanoparticle-filled polydimethylsiloxane (PDMS)-based mixed-matrix membranes (MMMs) were prepared from tubular ceramic carriers to meet the requirements of industrial applications, which were better than ZIF-67/PDMS (Co-ZIF-8/PDMS) MMMs in overall performance for ethanol recovery from aqueous solutions. Their separation factor for pervaporation was greatly enhanced compared with the pristine PDMS membrane; however, the flux reduction was slightly larger (flux-separation factor trade-off effect). Furthermore, Co_50_Zn_50_-ZIF-8/PDMS MMM was prepared through the substitution of Zn with Co in the Zn-ZIF-8 framework filler. Zn-ZIF-8 and Co_50_Zn_50_-ZIF-8 nanoparticles were studied using various characterizations, and the morphology and properties of the resulting MMMs were investigated. Owing to the relatively small agglomerations and enhancement of the pore volume of bimetallic Co_50_Zn_50_-ZIF-8 nanoparticles, the separation factor of Co_50_Zn_50_-ZIF-8/PDMS MMM was mostly similar to that of the Zn-ZIF-8/PDMS MMM, and the total flux was elevated, achieving the optimization of membrane properties. The best pervaporation performance of the Co_50_Zn_50_-ZIF-8/PDMS MMM was obtained in a 5 wt% ethanol aqueous solution at 60 °C with a total flux of 1.37 kg m^−2^ h^−1^, a corresponding separation factor of 10.5, and a pervaporation separation index (PSI) of 13.02 kg m^−2^ h^−1^. Overall, the relevant experimental results preliminarily demonstrated the potential application of Co_50_Zn_50_-ZIF-8/PDMS MMM in the separation of low-concentration ethanol aqueous solutions.

## Abstract 2

In the present investigation, highly hydrophobic Co_50_Zn_50_-ZIF-8 nanoparticle-filled polydimethylsiloxane (PDMS)-based mixed-matrix membranes (MMMs) were prepared from tubular ceramic carriers to meet the requirements of industrial applications.

## Graphical abstract

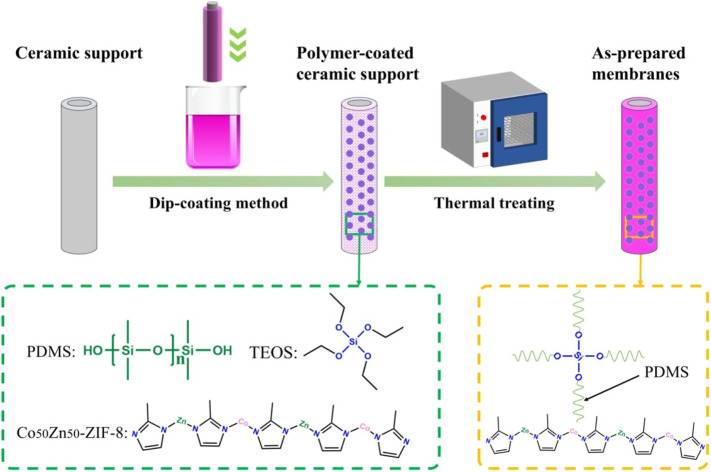

## 1. Introduction

Renewable biomass has attracted widespread attention as an effective alternative resource to conventional fossil fuels that cause energy depletion and environmental degradation.^1–3^ In particular, bioethanol, derived from the biomass fermentation of food and crop residues, has the peculiarity of sustainability and pollution-free combustion.^4–6^ However, the severe suppression of the end-product in the fermentation broth makes ethanol recovery from reaction mixtures a challenge.^7^ Currently, conventional distillation techniques are mostly applied in this purification process; nevertheless, high energy consumption and inefficient separation of the near-boiling systems pose certain challenges.^8,9^ In contrast to the traditional approaches, membrane-based pervaporation (PV) is considered a promising technology for ethanol recovery owing to its simple membrane treatment, low energy consumption, high selectivity and microbial non-toxicity.^10–12^

A suitable high-performance membrane is the core of PV, which is governed by the solution–diffusion mechanism, realizing the separation process through differences in the solubility and diffusion rates of the components in the membrane.^13^ Among the various membrane materials, PDMS has long been deemed as an ideal PV technology membrane by virtue of its excellent combination of advantages, such as strong hydrophobicity, good permeability and stability, and low production cost. However, the pristine PDMS membrane usually suffers from low separation performance and permeability–selectivity trade-off effects.^14,15^ For improving the permeation selectivity and reducing the trade-off limitation, the strategy of mixed-matrix membranes based on PDMS polymer matrices with filler incorporation is found to be effective.^16–18^ Nanoporous particles can be utilized as dopant fillers by enhancing selective diffusion for their rigid and porous structures to promote the membrane separation performance.^19,20^

Metal–organic frameworks (MOFs) employed as a new class of doped fillers have recently garnered considerable attention in the field of MMMs owing to their unique porous structures such as ZIF-8,^15^ ZIF-71,^21^ ZIF-67,^16^ MIL-53 (ref. 22) and UiO-66.^23^ Among them, zeolitic imidazolate framework-8 (ZIF-8), obtained by bridging the zinc cation with 2-methylimidazolite ions to realize a square natrium topology, has high porosities, excellent hydrophobicity and superior molecular adsorption, and these will preferentially allow the diffusion of ethanol molecules through their pore canal rather than water molecules.^24,25^ The alcoholophilicity is more prominent than that of other MOF particles (ZIF-67, MIL-53, and UiO-66). Moreover, the synthesis at room temperature of ZIF is more convenient compared with the synthesis condition of hydrothermal such as zeolite.^26–28^ Consequently, ZIF-8 particles have been partially applied and researched as more ideal doping fillers for MMMs, for example, Wang *et al.* designed covalently linked ZIF-8@PDMS MMMs and found that the total flux of the 7 wt%-loaded MMM reached 0.59 kg m^−2^ h^−1^ for the separation of 5 wt% ethanol aqueous solution, which increased by 34% compared to the PDMS membrane.^29^

To date, a large number of studies highlighting the synthesis of MMMs and the separation of ethanol aqueous solutions focus on the use of sheet carriers as supports, while tubular ceramic carriers are still lacking. On the contrary, tubular membranes are carried out in most of the industrial production, and tubular ceramics are currently the preferred carriers for commercial application due to their advantages for low transport resistance, high mechanical strength and inexpensiveness.^30,31^ In our previous research, a hydrophobic ZIF-67/PDMS (Co-ZIF-8 with no Zn content) MMM employing ceramic tubes as supports was fabricated, but the improvement of PV performance was not so desirable. In this work, a high hydrophobic ZIF-8/PDMS (Zn-ZIF-8 with no Co content) MMM was fabricated *via* incorporating Zn-ZIF-8 nanoparticles into a PDMS matrix on ceramic tube carriers. Its separation factor for PV performance was greatly enhanced compared with the pristine PDMS membrane; however, the flux reduction was slightly larger (a certain degree of flux-separation factor trade-off effect). Motivated on aforementioned researches, we further combined ZIF-8 with ZIF-67, that was the resulting for cobalt substituting Zn-ZIF-8 framework filler, the novel Co_50_Zn_50_-ZIF-8/PDMS MMM was prepared. Consequently, the modulation of cobalt substitution in the Zn-ZIF-8 framework to optimize the MMM separation performance was investigated in this study. The morphology and properties of Zn-ZIF-8 and Co_50_Zn_50_-ZIF-8 nanoparticles as well as the resulting MMMs were studied by various characterizations. Finally, the PV performance of the pristine PDMS membrane, Zn-ZIF-8/PDMS and Co_50_Zn_50_-ZIF-8/PDMS MMMs in ethanol recovery from aqueous solutions was evaluated: influence of loading, operating temperature and feed solution concentration on the membrane separation performance.

## 2. Experimental

### 2.1 Materials

Hydroxyl-terminated polydimethylsiloxane (PDMS, 5000 Pa s, molecular weight: 60 000, hydroxyl content: 0.1–0.5%) was purchased from Shenzhen Jipeng Silicon Fluorine Material Co., Ltd. Tetraethyl orthosilicate (TEOS, ≥98%), dibutyltin dilaurate (DBTOL, ≥95%), *n*-heptane (≥99%), zinc nitrate hexahydrate (Zn(NO_3_)_2_·6H_2_O, ≥99%), cobalt nitrate hexahydrate (Co(NO_3_)_2_·6H_2_O, ≥99%), 2-methylimidazole (Hmim, ≥99%), methanol (MeOH, ≥99%) and ethanol (EtOH, ≥99%) were obtained from Shanghai McLean Biochemical Technology Co., Ltd. The inexpensive α-alumina tube microporous supports with an average pore size of 200 nm used in this study were supplied by Foshan Ceramics Research Institute. The length, outer diameter, and inner diameter were 50 mm, 12 mm and 8 mm, respectively.

### 2.2 Membrane fabrication

Synthesis of Zn-ZIF-8 and bimetallic Co_50_Zn_50_-ZIF-8 nanoparticles: Zn-ZIF-8 was prepared following the procedure previously reported in the literature, with minor modifications, to synthesize a bimetallic Co–Zn-based organic framework.^32^ Initially, 0.21 g Zn(NO_3_)_2_·6H_2_O (and/or Co(NO_3_)_2_·6H_2_O; molar percentages of Co(NO_3_)_2_·6H_2_O used were 0%, 50%, and 100% to obtain Zn-ZIF-8, Co_50_Zn_50_-ZIF-8, and Co-ZIF-8 frameworks, respectively) and 0.48 g Hmim were dissolved in 11.80 g MeOH, respectively. Then, the two solutions were mixed under continuous stirring with a magnetic bar at room temperature to obtain white crystals. The precipitated product was collected *via* centrifugation at 8000 rpm for 10 min and washed repeatedly with methanol, and finally the particles were dried under vacuum at 80 °C overnight.

α-Alumina ceramic tube support treatment:^33^ The ceramic tubes were first polished with coarse (800 #) and fine (1500 #) sandpaper to increase the surface flatness, calcined to remove impurities and then cleaned through ultrasound. After that, they were immersed in pure water for 24 h, for the pores of carrier to be filled with water to prevent the coating polymer solution from penetrating into the pores. The carriers should be fetched out prior to impregnation with the coating polymer solution, 1 h in advance; then the surface was wiped with filter paper and left to dry for future use.

**Fig. 1:**
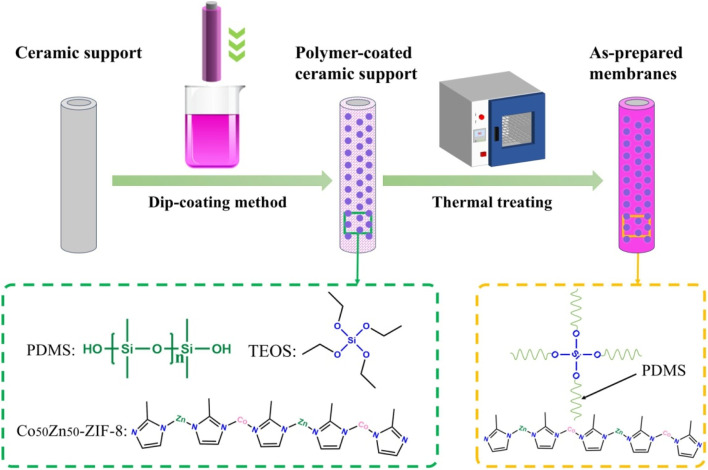
Schematic of the preparation of Co/Zn-ZIF/PDMS mixed-matrix membranes.

Preparation of PDMS MMMs: the schematic illustration of the Co/Zn-ZIF/PDMS mixed-matrix membrane preparation is presented in Fig. 1.^34^ An amount of filler particles (Zn-ZIF-8 and Co_50_Zn_50_-ZIF-8) were dispersed in 25 g *n*-heptane, stirred and ultrasonicated for 1 h to minimize aggregation. Then, a part of PDMS (10 wt% for the filler particles) were introduced and stirred for 4 h, and then, the remaining PDMS were added to the mixed solution and stirred for 12 h; the total mass added was 1.75 g (5 wt% PDMS for *n*-heptane amount). The cross-linking agent TEOS and catalyst DBTOL were added into the dispersion with a *W*_PDMS_/*W*_TEOS_/*W*_DBTOL_ mass ratio of 1/0.1/0.05 and left to defoam.^35^ The particle-filled PDMS layers were applied to the outer surface of pretreated tubular ceramic carriers by a dip-coating method for 1 min. Subsequently, through standing at room temperature for 24 h and vacuum drying at 90 °C for another 12 h, the tubular mixed matrix membrane was finally fabricated. Similarly, the pristine PDMS membranes were prepared using the above-mentioned cross-linked PDMS solution without the hybridization of Zn-ZIF-8 or Co_50_Zn_50_-ZIF-8 particles. The particle loadings of these membranes were 0, 5, 10, 15, 20 and 25 wt% for PDMS, respectively, in this study.

### 2.3 Characterization

The characterization for the crystal structures of the particles and hybrid membranes were confirmed using an X-ray diffractometer (XRD, Empyrean sharp shadow series, PANalytical, Netherlands) with CuKα radiation, generated between 5 and 60°. The chemical composition of MOF particles and membranes was recorded by Fourier transform infrared spectroscopy (FT-IR, Nicolet iS10, Thermo Fisher Scientific, USA), and the wave number range was set as 4000–400 cm^−1^. A Scanning Electron Microscope (SEM, Mira 4, Tescan, Czech Republic) was applied to examine the morphologies of MOF particles and membranes, and the elemental composition was evaluated by electron-dispersive spectroscopy (EDS) combined with FESEM measurement. N_2_ isotherms (77 K) of Zn-ZIF-8 and Co_50_Zn_50_-ZIF-8 particles were carried out using Brunauer–Emmett–Teller instruments (BET, 3H-2000PM1, Best, China); meanwhile, the surface area, pore size and pore volume were analyzed. The hydrophobic measurements on membrane surfaces were performed using a Contact Angle Goniometer (CA, JC2000DM, Zhongchen Shanghai, China). The evaluation was repeated at three different locations for each sample to measure the average contact angle result.

### 2.4 Membrane swelling

The adsorption capacity of membranes in water and ethanol was examined by swelling analysis. The tubular membranes (PDMS and MMMs) were sealed at both ends and immersed in a particular solvent (pure water, pure ethanol, and 5 wt% ethanol aqueous solution) at 60 °C for 24 h after drying in an oven overnight to remove moisture. The membrane samples were then removed, excess solvent was wiped with filter paper from the surface, and the samples were weighed using an electronic balance. The entire operation was repeated several times until no change in weight before and after immersion in solvents was observed. The swelling degree was calculated using eqn (1):1
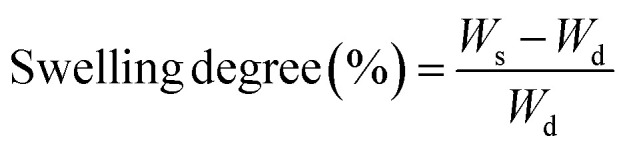
where *W*_d_ and *W*_s_ denote the weights of the initial and dissolved tubular membranes, respectively, g.

### 2.5 Pervaporation experiment

**Fig. 2:**
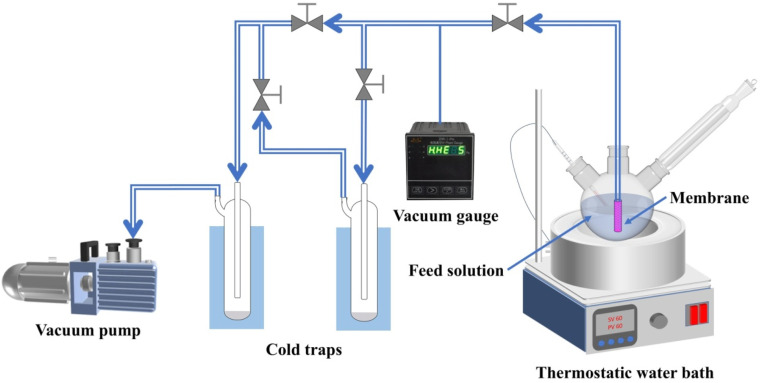
Schematic of the pervaporation performance test device.

The pervaporation evaluation was performed using a lab-scale homemade device, as shown in Fig. 2,^36,37^ employing an effective tubular membrane area for 16.95 cm^2^. The feed side was the solution for 5 wt% ethanol aqueous added to the 1-liter feed tank with the temperature controlled by a water bath at 60 °C, whereas the permeability side was under vacuum (less than 3 mbar) applying a vacuum pump. The collection for permeate began in a cold trap submerged in liquid nitrogen after 2 h of the piping system preheating. At the end of the experiment, the collected permeate samples were weighed and analyzed by gas chromatography (GC, GC9790II, Fuli, China). The total flux (*J*, kg m^−2^ h^−1^) and partial flux (*J*_i_) of the permeate were calculated by eqn (2) and (3):2
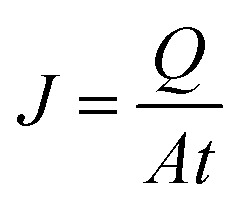

3 *J*_*i*_ = *y*_wi_*J*

where *Q* (kg) is the permeate total mass, *A* (m^2^) is the effective area for the membrane, *t* (h) is the permeate vaporization collection time, and *y*_wi_ is the mass fraction of component *i* in the permeate. The separation factor (*β*_*ij*_) was calculated by the ratio of the mass fractions for the two components on the permeate 
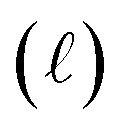
 and feed side (o), respectively, using eqn (4):4
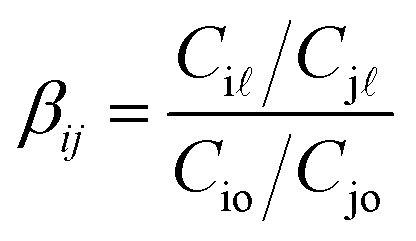


## 3. Results and discussions

### 3.1 Characterization of Zn-ZIF-8 and Co_50_Zn_50_-ZIF-8

**Fig. 3:**
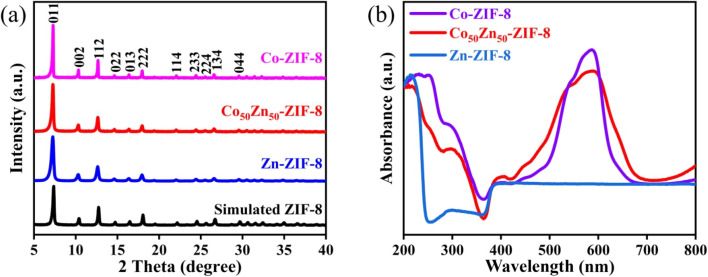
(a) XRD patterns and (b) UV-vis spectra of the bimetallic Co_50_Zn_50_-ZIF-8 and monometallic Zn-ZIF-8 and Co-ZIF-8 particles.

As illuminated in Fig. 3a, the synthesized bimetallic Co_50_Zn_50_-ZIF-8 and monometallic Zn-ZIF-8 and Co-ZIF-8 particles were characterized by XRD, wherein the characteristic diffraction peaks of Zn-ZIF-8 and Co-ZIF-8 particles are consistent with the spectra reported in the literature;^38^ meanwhile, the XRD patterns of Co_50_Zn_50_-ZIF-8 particles present peaks analogous to those of Zn-ZIF-8 and Co-ZIF-8. The results indicate that the bimetallic Co_50_Zn_50_-ZIF-8 has the sodalite topology adopting the same framework as Zn-ZIF-8, which is due to the quite comparable ionic radii for Zn^2+^ (0.74 Å) and Co^2+^ (0.72 Å) in tetrahedral coordination and the good interaction for Co substituting Zn ions.^39^ Additionally, a mild shift towards a lower 2*θ* value in the patterns of Co-ZIF-8 and Co_50_Zn_50_-ZIF-8 relative to Zn-ZIF-8 further supports the circumstance for Zn being substituted by Co ions in the Co_50_Zn_50_-ZIF-8 framework. Meanwhile, the UV/vis diffuse reflectance spectrum in Fig. 3b shows the feature bands of tetrahedral coordinated Co^2+^ at 588 nm and 540 nm,^40^ along with the characteristic bands of Zn-ZIF-8, which well demonstrates the construction of the Co_50_Zn_50_-ZIF-8 framework through the substitution of Zn by Co ions based on retaining the structural framework.

**Fig. 4:**
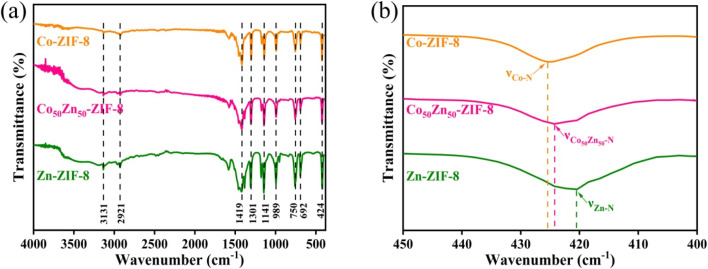
FT-IR spectra of Zn-ZIF-8, Co_50_Zn_50_-ZIF-8 and Co-ZIF-8 particles.

FTIR spectra were recorded to further confirm the functional groups of particles and the coexistence of Zn and Co in the bimetallic Co_50_Zn_50_-ZIF-8 framework, as displayed in Fig. 4a and b. The peaks in the range from 600 to 1500 cm^−1^ are assigned to the stretching and bending vibrations of the imidazole group,^41^ and the three particles of Co_50_Zn_50_-ZIF-8, Zn-ZIF-8 and Co-ZIF-8 are analogous at this point. In Fig. 4b, the stretching vibration modes of metal–nitrogen (M–N) at 424 cm^−1^ embodied the ZIF structure for the coordination of metal and Hmim ligands.^42^ Moreover, it is worth noting that the stretching of Zn/Co–N at 424 cm^−1^ is between Zn–N at 421 cm^−1^ and Co–N at 425 cm^−1^ through the comparison of the infrared bands for Co_50_Zn_50_-ZIF-8, Zn-ZIF-8 and Co-ZIF-8 particles, which affirms the coexistence of Zn and Co in the Co_50_Zn_50_-ZIF-8 framework.

**Fig. 5:**
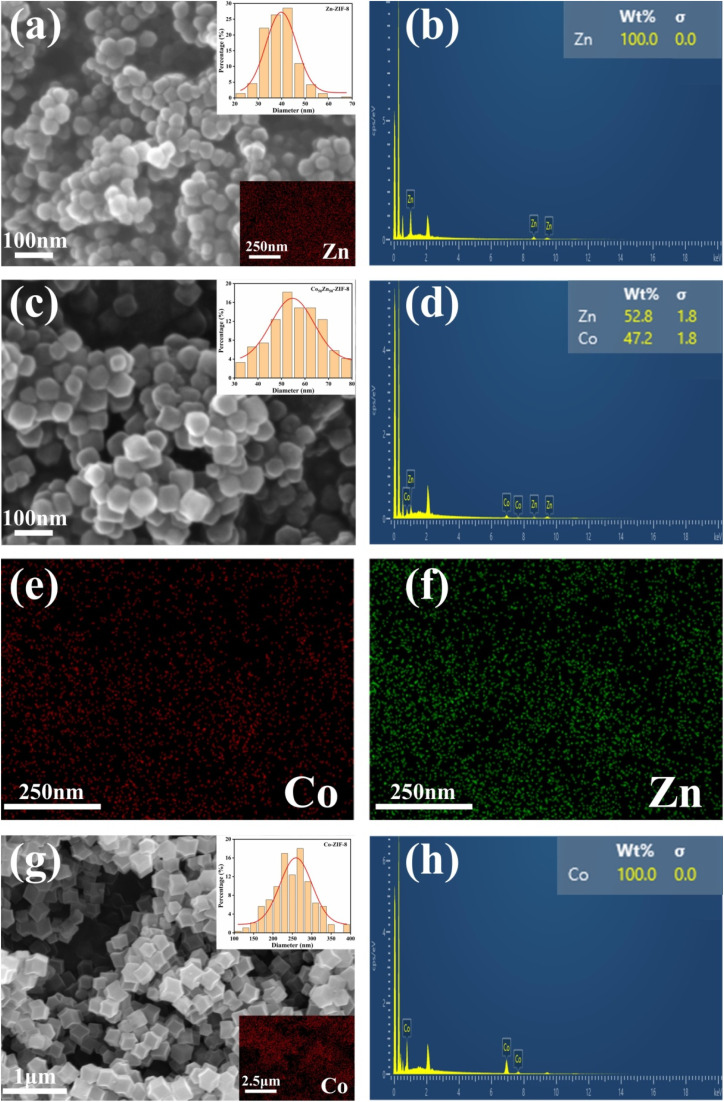
SEM images with particle size distribution histogram (inset) and elemental mapping (inset lower right) of (a) Zn-ZIF-8, (c) Co_50_Zn_50_-ZIF-8 (elemental mapping provided in (e and f)) and (g) Co-ZIF-8. EDS spectra for (b) Zn-ZIF-8, (d) Co_50_Zn_50_-ZIF-8 and (h) Co-ZIF-8. (e and f) Elemental mappings of Co_50_Zn_50_-ZIF-8.

In Fig. 5, the SEM images of the synthesized Co_50_Zn_50_-ZIF-8, Zn-ZIF-8 and Co-ZIF-8 show that the crystallized rhombic dodecahedral morphology of the Co/Zn-ZIF-8 framework. The particle size distribution in the inset suggests that the Zn-ZIF-8 particles are mainly distributed around 40 nm in size, and the size of Co_50_Zn_50_-ZIF-8 increases slightly to around 60 nm with Co substitution, both belonging to nanoparticles. However, the Co-ZIF-8 particles (ZIF-67, synthesized in our previous study) with the complete substitution of Zn by Co are much larger than them, reaching about 200–300 nm. In addition, the EDS results of Fig. 5d are in general agreement with the expected molar ratio of Co and Zn in the synthesized Co_50_Zn_50_-ZIF-8; meanwhile, elemental mapping in Fig. 5e and f reveals the uniform distribution of Co and Zn in the CoZn-ZIF-8 framework, which is well illustrated through the successful synthesis of Co_50_Zn_50_-ZIF-8 particles, consistent with the above-mentioned characterization results.

**Fig. 6:**
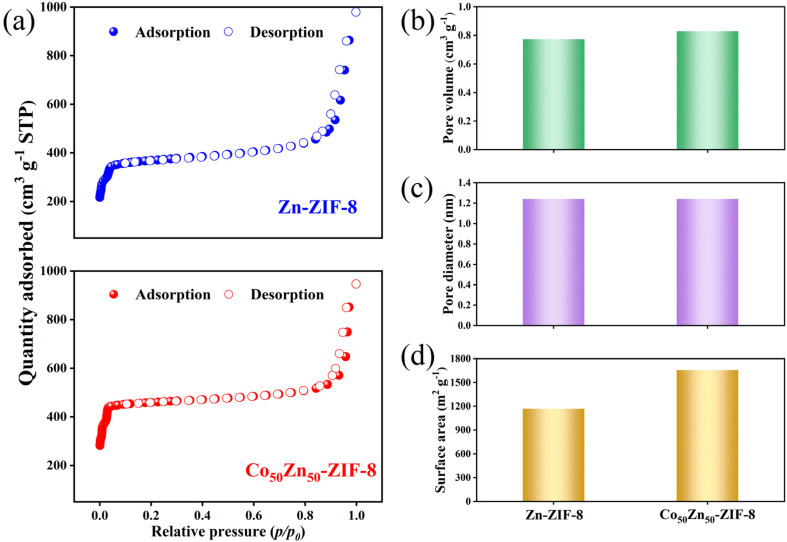
(a) Nitrogen adsorption–desorption isotherms, (b) pore volume, (c) pore diameter and (d) surface area of Zn-ZIF-8 and Co_50_Zn_50_-ZIF-8 particles.

Fig. 6 demonstrates the nitrogen isotherm at 77 K of Zn-ZIF-8 and Co_50_Zn_50_-ZIF-8 particles accompanied by their pore volume, pore size and surface area determined through the Brunauer–Emmett–Teller (BET) theory. The pore sizes of two particles remain essentially unchanged for 1.24 nm, indicating that they are microporous materials in nature. Nevertheless, the N_2_ adsorption–desorption isotherms both show the slight curve hysteresis, which is not consistent with the isotherm of microporous material, may be due to the small size of particles leading to slight agglomerates with stacking holes.^43^ Higher surface areas for Co_50_Zn_50_-ZIF-8 (1655 m^2^ g^−1^) than that of analogously synthesized Zn-ZIF-8 (1168 m^2^ g^−1^) suggests a smaller degree of agglomeration for Co_50_Zn_50_-ZIF-8 with the particle size slightly larger, which is in agreement with the surface SEMs of MMMs. The pore volumes increase from 0.82 to 0.77 cm^3^ g^−1^ with Co substituting Zn in the Zn-ZIF-8 framework, and this will promote more efficient diffusion pathways for feedstock.

### 3.2 Characterization of membrane materials

**Fig. 7:**
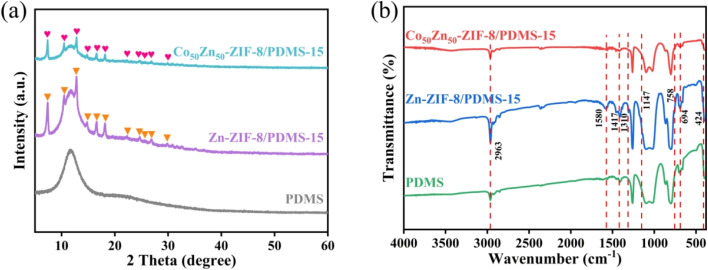
(a) XRD patterns and (b) FT-IR spectra of pristine PDMS membrane, Zn-ZIF-8/PDMS-15 wt% and Co_50_Zn_50_-ZIF-8/PDMS-15 wt% MMMs.

For membrane characterization, first, the structures of MMMs were investigated by XRD. Fig. 7a shows that a broad peak at 11.8° appears in the XRD patterns of all three membrane materials, attributed to the wide peak of amorphous features for the PDMS matrix.^44^ In addition, diffraction peaks of Co/Zn-ZIF-8 framework particles (red and orange markings in Fig. 7a) are also observed simultaneously in the diffractograms of Zn-ZIF-8/PDMS-15 wt% and Co_50_Zn_50_-ZIF-8/PDMS-15 wt% MMMs (the peak positions matched with the XRD patterns in Fig. 3a). These results indicated that Zn-ZIF-8 and Co_50_Zn_50_-ZIF-8 particles were successfully doped into PDMS matrix membranes as fillers and the structures in MMMs were not altered. Further FTIR spectra in Fig. 7b were obtained to confirm the chemical structure of the membrane materials. It is indicated that the infrared bands of the synthesized PDMS membrane contain strong peaks at 800 and 1260 cm^−1^, which accords with the characteristic distribution of PDMS infrared spectra, and the asymmetric stretching of Si–CH_3_ in PDMS is a reflection of the characteristic peak at 1030 cm^−1^.^45^ For Zn-ZIF-8/PDMS-15 wt% and Co_50_Zn_50_-ZIF-8/PDMS-15 wt% MMMs, the new peaks not present for pristine PDMS are consistent with the infrared absorption peaks of Co/Zn-ZIF-8 particles in Fig. 4b. These phenomena for infrared are associated with the XRD results.

**Fig. 8:**
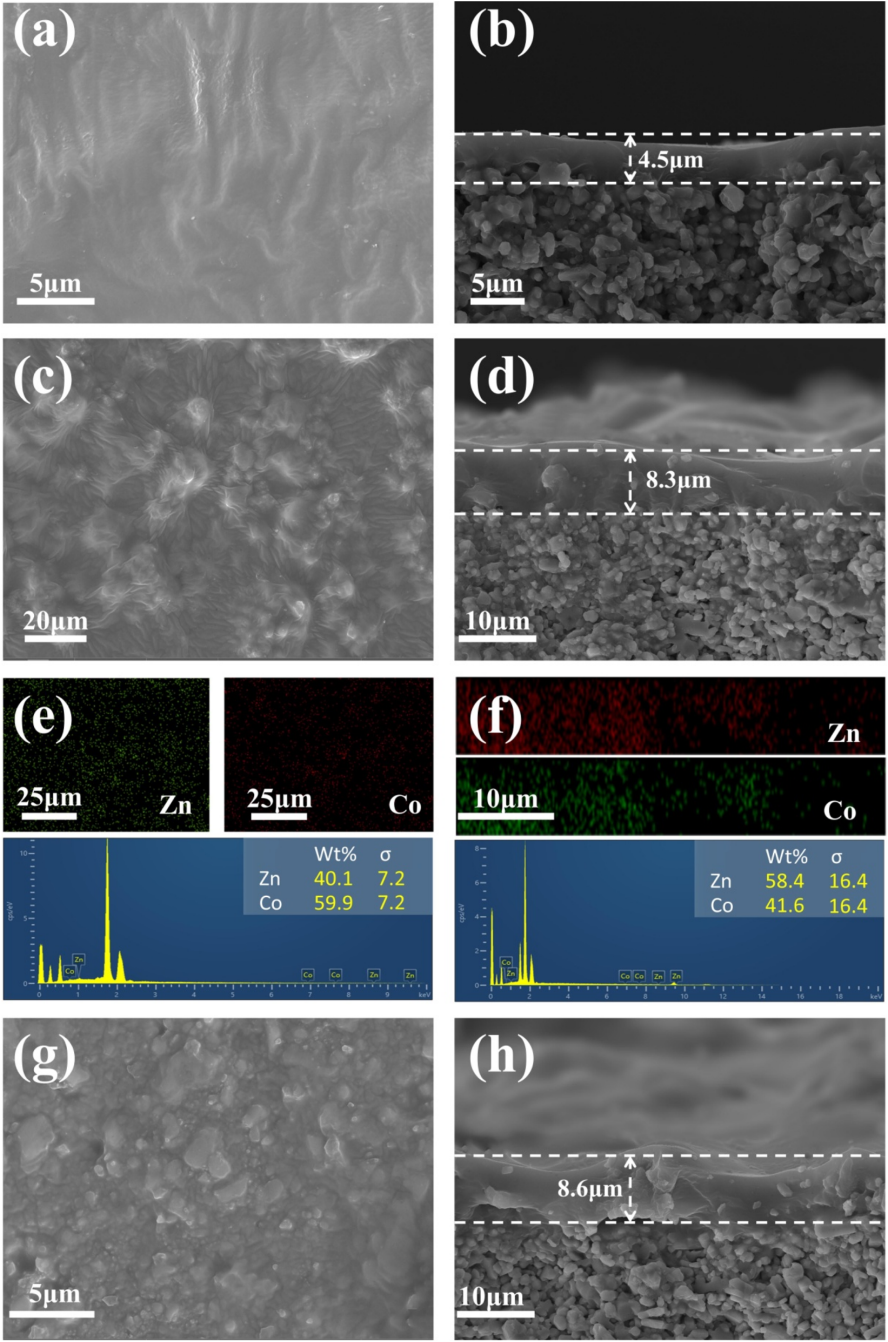
SEM morphologies for surfaces and cross-sections of (a and b) pristine PDMS membrane, (c and d) Co_50_Zn_50_-ZIF-8/PDMS-15 wt% MMM and (g and h) Zn-ZIF-8/PDMS-15 wt% MMM, and elemental mapping and EDS spectra for (e) surfaces and (f) cross-sections of Co_50_Zn_50_-ZIF-8/PDMS-15 wt% MMM.

The microscopic morphology and growth of the membranes were further examined by SEM. Fig. 8a and b exhibit that the pristine PDMS membrane has a dense, smooth and defect-free surface morphology with a film thickness of about 4.5 μm or so. As can be seen from the surface morphology of the ZIF-MMMs (Fig. 8c and g), the surfaces of MMMs doped with Co_50_Zn_50_-ZIF-8 and Zn-ZIF-8 particles become roughened, which is consistent with the results that the water contact angle on the surface of MMMs becomes larger after doping the fillers. Notably, the Zn-ZIF-8/PDMS MMM suffers from some degree of particle agglomeration (Fig. 8g and h); in contrast, less agglomeration is observed in the Co_50_Zn_50_-ZIF-8/PDMS MMM with a slightly larger size of filler particle, which will promote more efficient diffusion pathways for feedstock. Fig. 8e and f are correlated with the elemental mapping and EDS spectra for Co_50_Zn_50_-ZIF-8 (Fig. 5) and the XRD patterns for Co_50_Zn_50_-ZIF-8/PDMS-15 wt% MMM (Fig. 7), showing that the dopant fillers are indeed Co_50_Zn_50_-ZIF-8 particles and have been doped into the membrane. The average thickness of the ZIF-MMM selective film is about 8 μm, which is thicker than that of the pristine PDMS film, and there is no obvious peeling from the support layer.

**Fig. 9:**
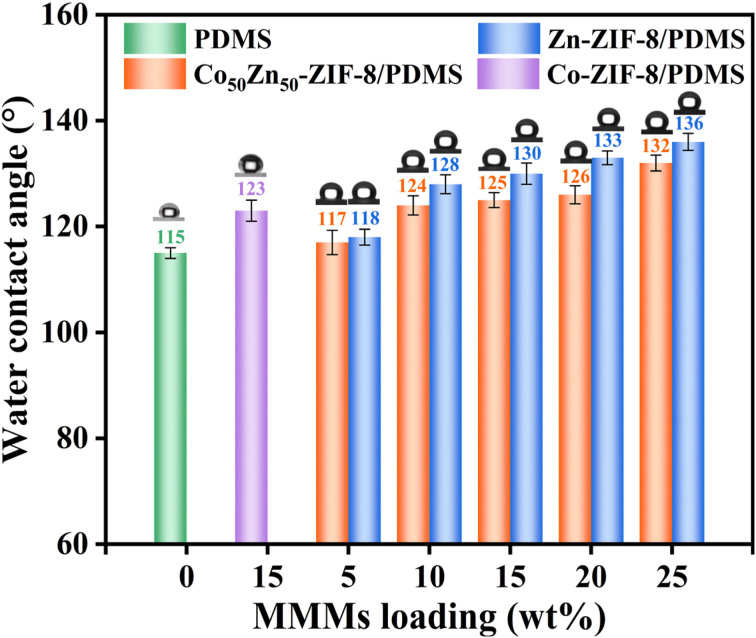
Water contact angles of Zn-ZIF-8/PDMS and Co_50_Zn_50_-ZIF-8/PDMS MMMs with different particle loading levels, pristine PDMS membrane and Co-ZIF-8/PDMS-15 wt% MMM.

The surface hydrophobicity of membranes is an important reference for their selective separability from aqueous ethanol solutions, which can be assessed by water contact angle measurements. As can be seen in Fig. 9, the water contact angles of the Zn-ZIF-8/PDMS and Co_50_Zn_50_-ZIF-8/PDMS MMMs are always higher than that of the pristine PDMS membrane (115°), which is attributed to the introduction of Zn-ZIF-8 and Co_50_Zn_50_-ZIF-8 fillers with preferable hydrophobicity, as well as an increase in the roughness of MMM surface. Owing to the better hydrophobicity of Zn-ZIF-8 particles, the water contact angle of the Zn-ZIF-8/PDMS MMM is somewhat higher than that of the Co_50_Zn_50_-ZIF-8/PDMS MMM at each loading concentration, and both of them are higher than that of the Co-ZIF-8/PDMS MMM (ZIF-67/PDMS MMM, synthesized in our previous study). The loading of fillers from 5% to 25% consistently enhanced the water contact angle of the MMMs due to the fact that the roughness of membrane surface elevated with the increase in doping content.

**Fig. 10:**
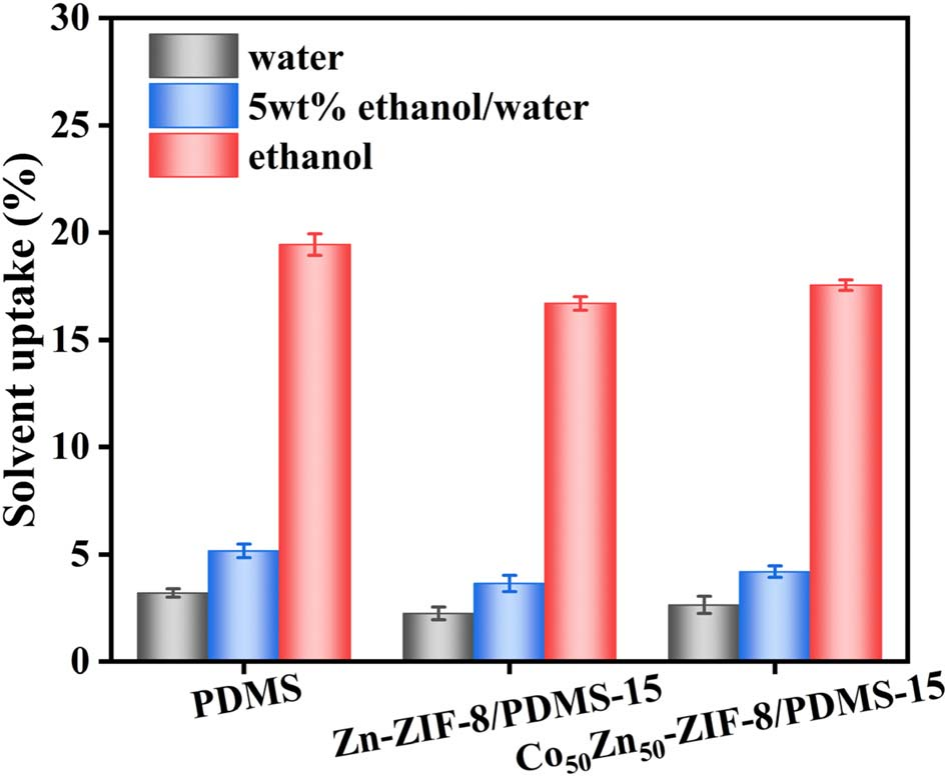
Swelling degree of pristine PDMS membrane, Zn-ZIF-8/PDMS-15 wt% and Co_50_Zn_50_-ZIF-8/PDMS-15 wt% MMMs in various solutions.

**Fig. 11:**
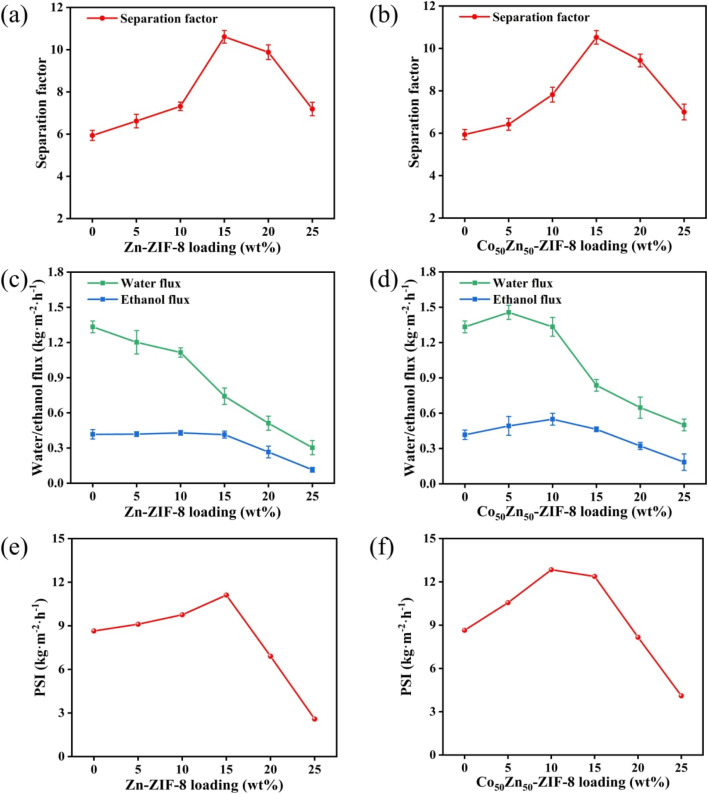
Effect of different Zn-ZIF-8 and Co_50_Zn_50_-ZIF-8 loading on (a and b) pervaporation performance, (c and d) ethanol flux and water flux, and (e and f) pervaporation separation index of MMMs (operating conditions: 5.0 wt% ethanol aqueous solution, 60 °C).

To better evaluate the doping effect of Zn-ZIF-8 and Co_50_Zn_50_-ZIF-8 particulate fillers, the swelling degree of the membranes in pure ethanol, 5 wt% ethanol aqueous solution and pure water at 60 °C was measured. As shown in Fig. 10, all three membranes exhibit the highest degree of adsorption in pure ethanol compared to that in a 5 wt% aqueous ethanol solution and pure water, indicating their better adsorption selectivity for ethanol molecules. This can be due to the fact that the Hildebrand solubility parameter of PDMS (15.5 MPa^1/2^) is closer to that of anhydrous ethanol (26.5 MPa^1/2^) rather than that of pure water (47.8 MPa^1/2^), whereas the MMMs possess hydrophobic inner channels of Zn-ZIF-8 and Co_50_Zn_50_-ZIF-8, acting as “solvent reservoir” toward ethanol molecules. However, through comparison, it can be seen that the pristine PDMS film displays higher solubility than Zn-ZIF-8/PDMS and Co_50_Zn_50_-ZIF-8/PDMS MMMs in the three solutions. It may be due to the case that the tube shape carrier leads to more or less agglomeration of the dopant particles, such that the filler particles in the matrix induce swelling stabilization at the polymer–filler interface, which reduces the void space of uptake.^11,16^ This also determines that the permeation fluxes of MMMs are lower than the result of the pristine PDMS membrane in the practical application of pervaporation for the separation of ethanol aqueous solution, in agreement with the conclusion of the flux variation in Fig. 11. The swelling degree of the Co_50_Zn_50_-ZIF-8/PDMS MMM is higher than that of the Zn-ZIF-8/PDMS MMM, and the possible cause is that the relatively small agglomerations of bimetallic Co_50_Zn_50_-ZIF-8 particles compensate for the lack of weaker hydrophobicity than that of Zn-ZIF-8, and the increased pore volume facilitates more diffusion pathways for penetrant molecular adsorption. Based on the solvation–diffusion mechanism, the results support that the bimetallic synergistic effect for doped filler optimizes pore connectivity and provides more efficient paths for molecular diffusion, and as a result, the adsorption capacity of pure ethanol, pure water and 5 wt% ethanol aqueous solution for Co_50_Zn_50_-ZIF-8/PDMS MMM all increased, making for the final separation factor being basically similar, while the permeation flux increases, which is a strong indication of the change in the results for MMMs in Fig. 11.

### 3.3 PV performance of membranes

The pervaporation separation performance of ethanol aqueous solution for Zn-ZIF-8/PDMS and Co_50_Zn_50_-ZIF-8/PDMS MMMs with different filler loadings was further investigated. As displayed in Fig. 11a, c, e for Zn-ZIF-8/PDMS MMM, when the Zn-ZIF-8 contents are increased from 0 to 25 wt%, the permeation flux decreases from 1.75 to 0.42 kg m^−2^ h^−1^. The phenomenon can be attributed to the following mechanisms: (1) the curved geometrical property of the tubular carrier leads to the formation of localized enriched zones for the fillers in the PDMS matrix (aggregation density >60% at loadings >15 wt%), which increases the tortuosity of mass transfer pathway; in contrast, the preparation of MMMs on sheet carriers is reported in the literature:^46^ scraping the mixed solution with a spatula on a sheet substrate at a thickness of 150 μm results in a more homogeneous dispersion of particles; (2) the submicron-sized Zn-ZIF-8 nanoparticles (average particle size ≈40 nm) exacerbate irreversible agglomeration dominated by van der Waals forces, consequently resulting in a gradual flux decline that remains consistently lower than that of the pristine PDMS membrane. Although the Co_50_Zn_50_-ZIF-8/PDMS system demonstrated a similar trend, its flux decay slope (−0.052 kg m^−2^ h^−1^/wt%) exhibited a reduction for 22% compared to the Zn-ZIF-8/PDMS system (−0.067 kg m^−2^ h^−1^/wt%). This indicates that particle size modulation (40 → 60 nm) of the bimetallic filler effectively mitigates interfacial defect issues, likely attributed to improved dispersion stability and optimized filler–matrix interactions. As for the separation factor, it increases from 5.9 to 10.6 to reach the highest with the increasing of particle loadings, which are all higher than those of the pristine PDMS membranes. Due to the strong hydrophobicity of Zn-ZIF-8 particles, the elevation of the hydrophobicity for composite membrane promotes the separation factor gradually increasing. However, as the Zn-ZIF-8 loading increases in the internal of 15–25 wt%, the separation factor begins to decrease, it may be the reason that the higher filler loadings result in severe agglomeration causing more interfacial defects between Zn-ZIF-8 fillers and PDMS matrix. Overall, the separation factor was greatly improved through doping the hydrophobic Zn-ZIF-8 particles compared with pristine PDMS membrane, but the flux was slightly larger reduced and there was a certain degree of flux-separation factor trade-off effect.

With Co substituting Zn in the Zn-ZIF-8 framework, the pervaporation performance of Co_50_Zn_50_-ZIF-8/PDMS MMMs at different loadings is shown in Fig. 11b, d, f. Similarly, the separation factor elevates with the increase in filler loadings and begins to decrease after reaching a maximum value of 10.5 at 15 wt%. Compared to the Zn-ZIF-8/PDMS MMM, there was a small reduction in the separation factor per concentration, but mainly similar. The decrease from 1.75 to 0.68 kg m^−2^ h^−1^ also shows a gradual decrease in flux, and all of them are lower than that of the PDMS membrane, but higher than that of the Zn-ZIF-8/PDMS MMM. Comprehensively, Co_50_Zn_50_-ZIF-8/PDMS MMM yields the highest PSI of 13.02 kg m^−2^ h^−1^.

**Fig. 12:**
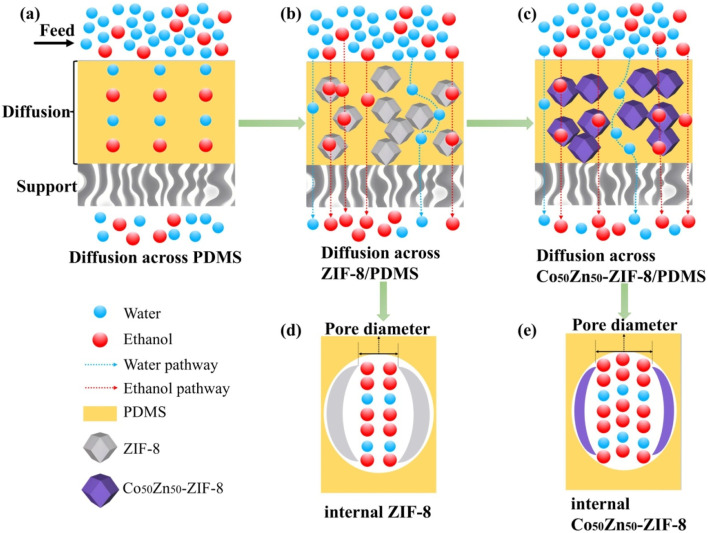
Schematic of the pristine PDMS membrane and MMM structure, and the main diffusion path of penetrant molecules: (a) pristine PDMS membrane; (b and d) Zn-ZIF-8/PDMS MMM; and (c and e) Co_50_Zn_50_-ZIF-8/PDMS MMM.

The aforementioned results can be interpreted based on the pervaporation mechanism model illustrated in Fig. 12. Although the hydrophobicity of Co_50_Zn_50_-ZIF-8 filler decreases compared to Zn-ZIF-8 (as evidenced by the differential water molecule transport pathways shown in blue in Schematics b and c), their larger particle size effectively suppresses the agglomeration for fillers (corresponding to the homogeneous distribution of purple polyhedrons in Schematic c), thereby maintaining comparable separation factors between Co_50_Zn_50_-ZIF-8/PDMS and Zn-ZIF-8/PDMS MMMs. More critically, the isomorphous substitution of Zn with Co in the Zn-ZIF-8 framework induces significant pore volume expansion (as demonstrated by the contrasting pore profiles in Schematics d and e, where the latter exhibited dual-molecule transport channels), and this enlarged pore architecture provides enhanced permeation pathways for water/ethanol molecules. Following the solution-diffusion mechanism, the bimetallic synergy in doped fillers optimizes pore connectivity, establishing more efficient molecular diffusion routes; consequently, the Co_50_Zn_50_-ZIF-8/PDMS MMM demonstrates increased adsorption capacity for ethanol aqueous solutions, ultimately achieving 18.7% enhancement for flux. These findings validate the dual optimization mechanism of filler topology regulation on membrane separation performance: by balancing filler dispersibility with pore characteristics, this strategy not only preserves separation selectivity but also overcomes the permeability limitations inherent in conventional membranes.

**Fig. 13:**
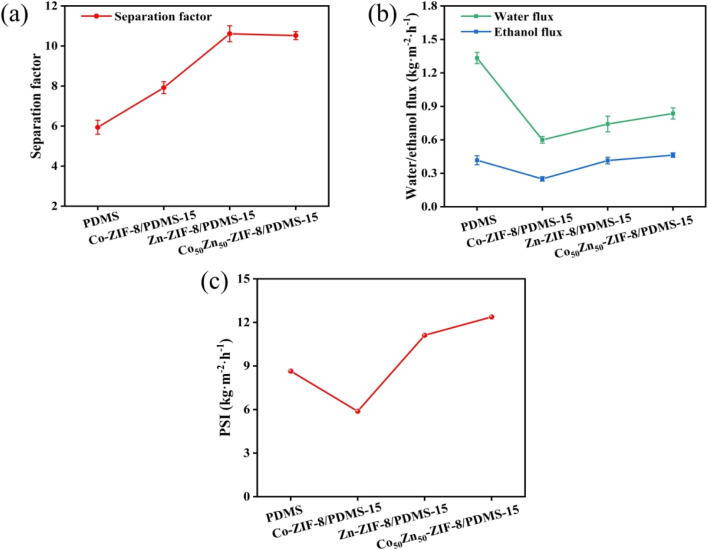
(a) Pervaporation performance, (b) ethanol flux and water flux, and (c) pervaporation separation index of pristine PDMS membrane, and Co-ZIF-8/PDMS-15 wt%, Zn-ZIF-8/PDMS-15 wt% and Co_50_Zn_50_-ZIF-8/PDMS-15 wt% MMMs (operating conditions: 5.0 wt% ethanol aqueous solution, 60 °C).

The pervaporation performances of the pristine PDMS membranes, Co-ZIF-8/PDMS-15 wt% (ZIF-67/PDMS, synthesized in our previous study), Zn-ZIF-8/PDMS-15 wt% and Co_50_Zn_50_-ZIF-8/PDMS-15 wt% MMMs for ethanol recovery are compared in Fig. 13. Through a comparative analysis of pervaporation performance across different systems, the MMMs with 15 wt% filler loading exhibits the following characteristics: (1) in terms of total flux, the MMMs in this work demonstrates intermediate between the pristine PDMS membrane (1.75 kg m^−2^ h^−1^) and Co-ZIF-8/PDMS MMM (0.85 kg m^−2^ h^−1^); (2) the bimetallic Co_50_Zn_50_-ZIF-8/PDMS system achieves optimal performance with ethanol flux of 0.55 kg m^−2^ h^−1^, representing 30.1% enhancement over the Zn-ZIF-8/PDMS system for 0.42 kg m^−2^ h^−1^; (3) regarding separation selectivity, both Zn-ZIF-8/PDMS and Co_50_Zn_50_-ZIF-8/PDMS MMMs exhibits comparable separation factors of approximately 10.6 and 10.5, respectively, these values correspond to 79.7% and 78.0% improvements relative to the pristine PDMS membrane (5.9), while significantly surpassing the Co-ZIF-8/PDMS MMM (7.9). Comprehensively, Co_50_Zn_50_-ZIF-8/PDMS MMM yields the highest PSI of 13.02 kg m^−2^ h^−1^ among the four membranes. It reveals that the bimetallic strategy effectively mitigates the traditional permeability-selectivity trade-off through precise regulation of filler particle size and pore architecture, while maintaining satisfactory separation selectivity.

### 3.4 Effect of operating conditions on the PV performance

#### 3.4.1 Effect of operating temperature on MMMs

**Fig. 14:**
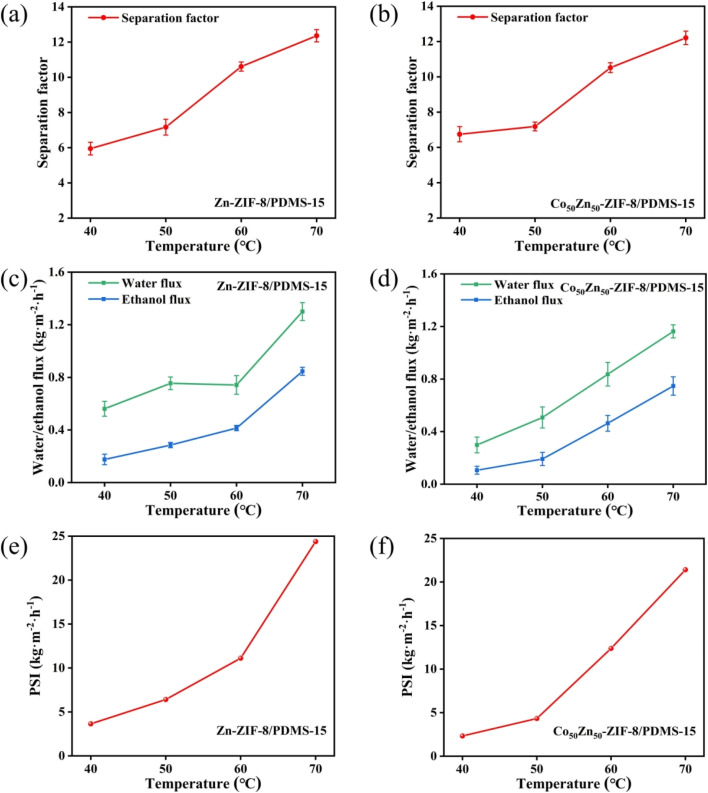
Effect of operating temperature on (a and b) pervaporation performance, (c and d) ethanol flux and water flux, and (e and f) pervaporation separation index of Zn-ZIF-8/PDMS-15 wt% and Co-ZIF-8/PDMS-15 wt% MMMs (operating conditions: 5.0 wt% ethanol aqueous solution).

**Fig. 15:**
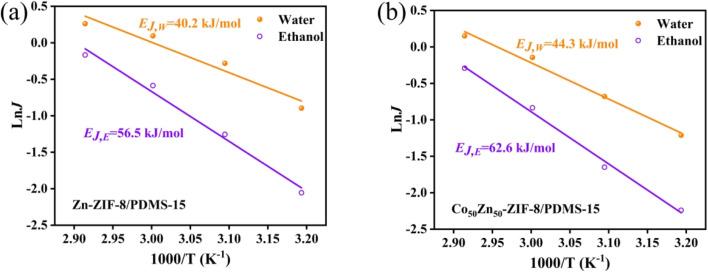
Arrhenius plots of the individual flux against the reciprocal temperature for (a) Zn-ZIF-8/PDMS-15 wt% and (b) Co-ZIF-8/PDMS-15 wt% MMMs.

The effects of operating temperature for ethanol recovery on the pervaporation performance for Zn-ZIF-8/PDMS and Co_50_Zn_50_-ZIF-8/PDMS MMMs are shown in Fig. 14. It can be seen that the total flux, partial flux and separation factor of Zn-ZIF-8/PDMS and Co_50_Zn_50_-ZIF-8/PDMS MMMs gradually increases with the increase in temperature, indicating that the temperature rising contributes to the dealcoholization performance of MMMs in terms of permeation flux and separation factor. As an activation process, the effect of feed temperature on the individual flux can be further described by the Arrhenius equation:^47^5
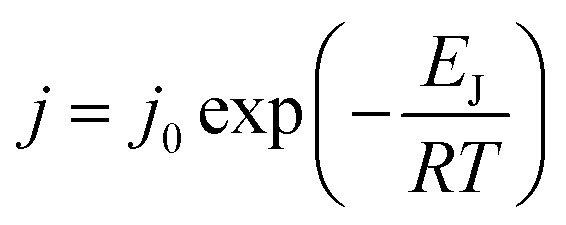
where *J*_0_ is the pre-exponential factor of flux, *R* is the ideal gas constant, and *T* is the operating temperature. *E*_J_ denotes the apparent activation energy of flux, which can be calculated from eqn (5). As shown in Fig. 15, the good linear fit indicated that the experimental data fit well into the Arrhenius equation and the apparent activation energy values of 56.5 kJ mol^−1^, 40.2 kJ mol^−1^ (Zn-ZIF-8/PDMS MMM) and 62.6 kJ mol^−1^, 44.3 kJ mol^−1^ (Co_50_Zn_50_-ZIF-8/PDMS MMM) were obtained for ethanol and water, respectively. The temperature increasing produced a higher difference of vapor pressure across membranes, which enhanced the transport driving force, resulting in a promotion in the diffusion rate of both ethanol and water molecules. The apparent activation energy of the ethanol flux was higher than that of the water flux, which demonstrated that the transport rate of ethanol molecules through the membranes was more sensitive to temperature, leading to a continuous elevation in the separation factor for dealcoholization with operating temperature rising.

The incorporation of Co into the ZIF-8 framework optimized the distribution of adsorption sites through electronic effects, resulting in an enhancement for 10.8% in ethanol activation energy and improved thermoresponsive characteristics. The analysis of vapor pressure gradient-driven mass transfer kinetics demonstrated that a temperature elevation for 10 °C induced an increase in ethanol flux by 18–22%, accompanied by a concomitant improvement of 12–15% in separation factor, and it collectively validated the dual enhancement effects of the thermal activation process on selective mass transport, simultaneously amplifying both permeation efficiency and molecular discrimination capability.

#### 3.4.2 Effect of feed concentration on MMMs

**Fig. 16:**
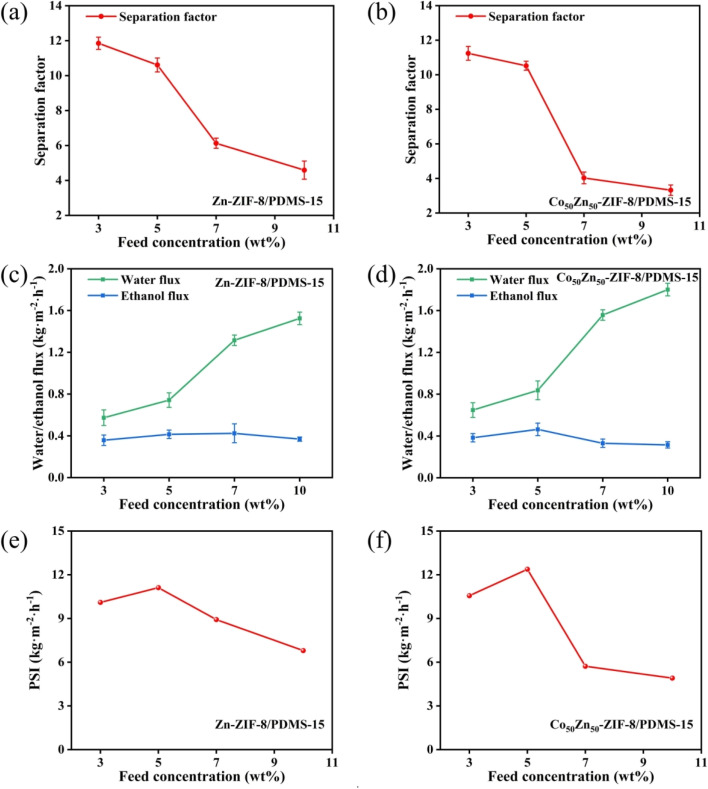
Effect of feed concentration on (a and b) pervaporation performance, (c and d) ethanol flux and water flux, and (e and f) pervaporation separation index of Zn-ZIF-8/PDMS-15 wt% and Co-ZIF-8/PDMS-15 wt% MMMs (operating temperature: 60 °C).

Fig. 16 displays the effect of feed concentration on the pervaporation performance for Zn-ZIF-8/PDMS and Co_50_Zn_50_-ZIF-8/PDMS MMMs. The total and partial fluxes of Zn-ZIF-8/PDMS and Co_50_Zn_50_-ZIF-8/PDMS MMMs are increasing with the increase in ethanol concentration in the feed, while the separation factor shows a trend for decreasing. The increase in feed concentration promoted the adsorption of ethanol on the membranes, which caused the PDMS matrix to become more swollen, and its segments had fuller volume freedom and mobility, as a result, the diffusion rate of the two permeate fractions were elevated, leading to an increase in total flux.^48^ In addition, the feed concentration rising makes more ethanol molecules, and the diameter of ethanol molecules is larger, which leads to an increment in the resistance for diffusion process, so that the diffusion rate of ethanol is lower than that of water, and the separation factor reduced. The concentration-dependent mass transfer competition mechanism revealed that topological optimization of ZIF fillers (modulation of pore-channel in bimetallic Co_50_Zn_50_-ZIF-8) enabled synergistic regulation of swelling-enhanced effect and size-sieving effect, thereby achieving a selectivity equilibrium under high permeation flux.

### 3.5 Performance benchmarking

The comparison of pervaporation properties for Zn-ZIF-8/PDMS and Co_50_Zn_50_-ZIF-8/PDMS MMMs with other PDMS-based MMMs filling different fillers in ethanol aqueous solutions is given in Table 1. Comparing the performance of these membranes is difficult due to different parameters such as the type, nature and loading for the filler, sharp and material for the support, and thickness of the selective layer. However, it can be inferred that the MMMs in this work have high separation factor, satisfactory total flux and high PSI among the representative state of the studies listed in Table 1. Moreover, the MMMs in this work were prepared with tubular ceramics, this improvement is further close to the requirement of industrialized application, and no found in other studies, which is the reason why the total flux of MMMs is decreased in the middle level. Therefore, for further comparison, our research team also synthesized silicalite-1/PDMS MMM with ceramic tubes as the carrier and silicalite-1 particles as the filler, and the comparison for series of membranes in the work of our research team shows that Co_50_Zn_50_-ZIF-8/PDMS MMM has better pervaporation properties. It can be said that the Co_50_Zn_50_-ZIF-8/PDMS MMM has a great potential for application in the pervaporation of ethanol recovery.

**Table 1:** Comparison of the pervaporation performance of PDMS-based MMMs in ethanol aqueous solutions

Membrane	Filler loading (wt%)	Feed temperature (°C)	Feed concentration (wt%)	Total flux (kg m^−2^ h^−1^)	Separation factor	PSI (kg m^−2^ h^−1^)	Ref.
S-ZIF-90/PDMS	15	40	5	1.06	14.9	14.73	19
F12/GZIF-8/PDMS	3	60	5	1.68	9.5	14.28	49
POSS-GO/PDMS	0.2	40	5	1.35	11.2	13.77	50
Silicalite-1/PDMDES	15	40	5	6.07	9.5	51.60	51
K-MWCNT/PDMS	2	50	6	0.98	10.4	9.21	45
ZIF-L/PDMS	15	40	5	1.12	12.3	12.66	52
UiO-66/α-Al_2_O_3_	—	50	10	1.40	4.9	5.46	53
PDMS	—	60	5	1.75	5.9	8.58	This work
Silicalite-1/PDMS	15	60	5	0.43	6.4	2.32	This work
Zn-ZIF-8/PDMS	15	60	5	1.15	10.6	11.04	This work
Co_50_Zn_50_-ZIF-8/PDMS	15	60	5	1.37	10.5	13.02	This work
Co-ZIF-8/PDMS	15	60	5	0.85	7.9	5.87	This work

## 4. Conclusions

In this work, with Zn-ZIF-8 and Co substituting Zn in Zn-ZIF-8 framework, Co_50_Zn_50_-ZIF-8 nanoparticles were embedded into a PDMS matrix on ceramic tube carriers to develop Zn-ZIF-8/PDMS and Co_50_Zn_50_-ZIF-8/PDMS MMMs for ethanol recovery from aqueous solutions *via* pervaporation. The XRD, UV and FTIR characterization of fillers confirmed the successful synthesis of Zn-ZIF-8 and Co_50_Zn_50_-ZIF-8 fillers. Then it was indicated that the Co_50_Zn_50_-ZIF-8 framework displayed the enhancement of surface area and pore volume as compared to the Zn-ZIF-8 framework from the results of BET. Meanwhile, the XRD pattern and FTIR spectra of MMMs demonstrated Zn-ZIF-8 and Co_50_Zn_50_-ZIF-8 particles successfully doping into PDMS matrix membranes as fillers, and the Zn-ZIF-8/PDMS MMM suffering from some degree of particle agglomeration and less agglomeration for Co_50_Zn_50_-ZIF-8/PDMS MMM were identified through SEM observation. Compared to the PDMS membranes, the doping of highly hydrophobic Zn-ZIF-8 resulted in a large enhancement of the separation factor for pervaporation of Zn-ZIF-8/PDMS MMM; however, the flux reduction is slightly larger. With Co substituting Zn in the Zn-ZIF-8 framework, the relatively small agglomerations of bimetallic Co_50_Zn_50_-ZIF-8 particles compensated for the lack of weaker hydrophobicity than that of Zn-ZIF-8 and kept the separation factor for the Co_50_Zn_50_-ZIF-8/PDMS MMM at essentially the same level as the Zn-ZIF-8/PDMS MMM; meanwhile, the increase in pore volume facilitated more diffusion pathways for penetrant molecules, leading to an elevation in the total flux for Co_50_Zn_50_-ZIF-8/PDMS MMM. The pervaporation performance of the Co_50_Zn_50_-ZIF-8/PDMS MMM with a loading of 15 wt% in a 5 wt% ethanol aqueous solution at 60 °C was the best, with a total flux of 1.37 kg m^−2^ h^−1^, a separation factor of 10.5 and a PSI of 13.02 kg m^−2^ h^−1^. Therefore, the Co_50_Zn_50_-ZIF-8/PDMS composite membrane with excellent properties is a promising candidate membrane material.

## Conflicts of interest

There are no conflicts to declare.

## Data Availability

The authors declare that the data supporting this manuscript are available in the article.
